# West Nile Virus Meningoencephalitis Imported into Germany

**DOI:** 10.3201/eid1810.120204

**Published:** 2012-10

**Authors:** Jörg Schultze-Amberger, Petra Emmerich, Stephan Günther, Jonas Schmidt-Chanasit

**Affiliations:** Klinikum Ernst von Bergmann, Potsdam, Germany (J. Schultze-Amberger);; and Bernhard Nocht Institute for Tropical Medicine, Hamburg, Germany (P. Emmerich, S. Günther, J. Schmidt-Chanasit)

**Keywords:** West Nile virus, arbovirus, Germany, serology, viruses, vector-borne infections

**To the Editor:** West Nile virus (WNV) is a single-stranded RNA virus in the family *Flaviviridae* that is transmitted to humans by mosquitoes. Approximately 80% of WNV infections in humans are asymptomatic, whereas ≈20% of infected persons experience fever, often accompanied by a rash. Less than 1% of infections are manifested as neuroinvasive disease, such as meningoencephalitis, polyradiculoneuritis, and polio-like flaccid paralysis (*1*). WNV is endemic in Africa, southern Asia, and northern Australia, and only sporadic cases or small epidemics are seen in Europe (*2*). In 1999, WNV emerged in North America. By 2010, ≈1.8 million persons had become infected, with 12,852 reported cases of meningoencephalitis and 1,308 deaths (*2*). In Europe, the last notable outbreak of WNV infection occurred in Greece in 2010; 197 persons were infected, and 33 died (*3*). The Czech Republic, Denmark, France, and the Netherlands reported laboratory-confirmed WNV infections in travelers returning from North America (*1*).

We report a case of WNV meningoencephalitis in a 28-year-old German woman, who sought treatment the emergency department of a hospital in Potsdam, Germany, on September 7, 2011. She had a 3-day history of fever of up to 40°C and mental confusion. Six days before admission, she had returned from a 2-week holiday trip to Ottawa, Ontario, Canada. She had spent most of her time in the city of Ottawa.

The patient’s medical history was unremarkable. She was in a reduced general condition because of a severe encephalitic syndrome characterized by somnolence, meningism, fever, and mental confusion. Laboratory investigations revealed leukocytosis with 15.000 leukocytes/µL (reference range 4,400–11,300 leukocytes/µL) and elevated C-reactive protein of 14.8 mg/L (reference <3 mg/L). Cerebrospinal fluid (CSF) analysis on the day of admission showed pleocytosis, 430 cells/µL (72% granulocytes, 27% lymphocytes. and 1% monocytes); elevated levels for total protein, 1,023 mg/L (reference range 150–450 mg/L); an albumin level of 637 mg/L (normal range 0–350 mg/L); and a moderately elevated level of the albumin quotient of 20 (reference range <6.5). The CSF/serum diagrams demonstrated a moderate disturbance of the blood–CSF barrier and a substantial intrathecal IgM synthesis of 27.6% (6.15 mg/L) but no intrathecal IgA or IgG synthesis. Results of magnetic resonance imaging of the brain were unremarkable. No parenchymal lesions were found.

Antimicrobial drug therapy was initiated with ceftriaxone and ampicillin. Acyclovir was administered empirically for herpes simplex encephalitis until this diagnosis was excluded. Molecular and serologic testing of serum and CSF samples revealed no acute infection with herpesviruses, enteroviruses, alphaviruses, orthobunyaviruses, and arenaviruses or with mycobacteria, *Borrelia* spp., *Toxoplasma gondii*, *Chlamydia* spp., *Leptospira* spp., and *Mycoplasma pneumoniae*. CSF and blood cultures were negative for fungi and bacteria, including mycobacteria. An encephalitic syndrome caused by N-methyl-D-aspartate antibodies was also excluded. On the basis of the patient’s travel history, the clinical symptoms, and the initial laboratory findings, WNV infection was suspected. Indirect immunofluorescence assays and virus neutralization tests (VNT) for WNV and other flaviviruses were performed as described (*4*). IgM and IgG against WNV were detected in serum and in CSF by indirect immunofluorescence assay with an 8-fold (IgM) and 32-fold (IgG) increase in serum titer from day 4 to day 26 (Table). WNV IgG and WNV IgM titers were higher than the titers of antibodies against the other flaviviruses tested (Table), indicating that the antibodies resulted from a WNV infection. The serologic diagnosis was further substantiated by detection of WNV neutralizing antibodies at day 11 (VNT titer 640). The VNT titer further increased to 2,560 on day 26 after onset of disease. Results of reverse transcription PCR were negative for WNV and members of genus *Flavivirus* in serum and CSF samples taken 4 days after disease onset. Attempts to isolate WNV from serum and CSF samples in cell culture failed as well. The patient recovered slowly and was discharged from the hospital in Potsdam on September 15, 2011. She was then referred to a neurologic rehabilitation center in Berlin and was discharged from there after 2 months with a characterization of *restitutio ad integrum* (i.e., full recovery, restoration to original condition).

**Table:** Results of indirect immunofluorescence assays performed on serum and CSF samples from patient with suspected WNV infection, Germany, 2011*

Virus used as antigen	Ig class	Antibody titer in serum					Antibody titer in CSF	
		Day 4	Day 6	Day 11	Day 26		Day 4	Day 6
WNV	IgG	320	1,280	5,120	10,240		20	40
WNV	IgM	160	160	1,280	1,280		10	20
SLEV	IgG	80	80	1,280	2,560		<10	<10
SLEV	IgM	20	20	40	<20		<10	<10
JEV	IgG	ND	<20	ND	1,280		ND	<10
JEV	IgM	ND	<20	ND	<20		ND	<10
DENV	IgG	ND	80	ND	640		ND	<10
DENV	IgM	ND	20	ND	<20		ND	<10
YFV	IgG	ND	<20	ND	ND		ND	<10
YFV	IgM	ND.	<20	ND	ND		ND	<10
TBEV	IgG	ND	<20	ND	ND		ND	<10
TBEV	IgM	ND	<20	ND	ND		ND	<10

*CSF, cerebrospinal fluid; WNV, West Nile virus; SLEV, St. Louis encephalitis virus; JEV, Japanese encephalitis virus; ; ND, not done; DENV, dengue virus; YFV, yellow fever virus; TBEV, tick-borne encephalitis virus. Titers <20 for serum and <10 for CSF are considered negative.

We report a case of WNV infection imported into Germany that was unambiguously confirmed by laboratory testing. WNV meningoencephalitis was diagnosed on the basis of strict serologic criteria established by the Centers for Disease Control and Prevention (Atlanta, GA, USA) (*5*). In 2011, 69 clinical cases of WNV infection were reported from the province of Ontario, although no cases in the city of Ottawa were reported (Public Health Agency of Canada; www.eidgis.com/wnvmonitorca). This imported case adds to these cases and suggests that travelers are at risk, even if they visit only Ottawa. Physicians in Germany should be aware of the risk for WNV infection among travelers returning from Canada, especially during late summer.
